# EIF3H promotes aggressiveness of esophageal squamous cell carcinoma by modulating Snail stability

**DOI:** 10.1186/s13046-020-01678-9

**Published:** 2020-08-31

**Authors:** Xiaobin Guo, Rui Zhu, Aiping Luo, Honghong Zhou, Fang Ding, Hongxin Yang, Zhihua Liu

**Affiliations:** 1 State Key Laboratory of Molecular Oncology, National Cancer Center/National Clinical Research Center for Cancer/Cancer Hospital, Chinese Academy of Medical Sciences and Peking Union Medical College, Beijing, 100021 PR China; 2 Department of Clinical pharmacy, Inner Mongolia Autonomous Region People’s Hospital, Hohhot, Inner Mongolia 010017 PR China

**Keywords:** EIF3H, Snail, Deubiquitination, Esophageal squamous cell carcinoma

## Abstract

**Background:**

Overexpression of eukaryotic translation initiation factor 3H (EIF3H) predicts cancer progression and poor prognosis, but the mechanism underlying EIF3H as an oncogene remains unclear in esophageal squamous cell carcinoma (ESCC).

**Methods:**

TCGA database and the immunohistochemistry (IHC) staining of ESCC samples were used and determined the upregulation of EIF3H in ESCC. CCK8 assay, colony formation assay and transwell assay were performed to examine the ability of cell proliferation and mobility in KYSE150 and KYSE510 cell lines with EIF3H overexpression or knockdown. Xenograft and tail-vein lung metastatic mouse models of KYSE150 cells with or without EIF3H knockdown were also used to confirm the function of EIF3H on tumor growth and metastasis in vivo. A potential substrate of EIF3H was screened by co-immunoprecipitation assay (co-IP) combined with mass spectrometry in HEK293T cells. Their interaction and co-localization were confirmed using reciprocal co-IP and immunofluorescence staining assay. The function of EIF3H on Snail ubiquitination and stability was demonstrated by the cycloheximide (CHX) pulse-chase assay and ubiquitination assay. The correlation of EIF3H and Snail in clinical ESCC samples was verified by IHC.

**Results:**

We found that EIF3H is significantly upregulated in esophageal cancer and ectopic expression of EIF3H in ESCC cell lines promotes cell proliferation, colony formation, migration and invasion. Conversely, genetic inhibition of EIF3H represses ESCC tumor growth and metastasis in vitro and in vivo. Moreover, we identified EIF3H as a novel deubiquitinating enzyme of Snail. We demonstrated that EIF3H interacts with and stabilizes Snail through deubiquitination. Therefore, EIF3H could promote Snail-mediated EMT process in ESCC. In clinical ESCC samples, there is also a positive correlation between EIF3H and Snail expression.

**Conclusions:**

Our study reveals a critical EIF3H-Snail signaling axis in tumor aggressiveness in ESCC and provides EIF3H as a promising biomarker for ESCC treatment.

## Background

Esophageal cancer is the 7th most common cancer and the 6th most common cause of death from cancer worldwide, with an approximate 572,000 new cases and 509,000 deaths in 2018 [1]. Esophageal squamous cell carcinoma (ESCC) accounts for about 90% cases of esophageal cancer worldwide [2], and more than 50% of ESCC occur in China [3]. Esophageal cancer has a very poor five-year survival rate below 30% [4], mainly because of limited clinical approaches for early diagnosis, tumor metastasis before diagnosis and tumor recurrence [5–7]. However, molecular mechanisms underlying these processes are still not clear. Thus, a compelling study is required for the better understanding of the molecular basis of ESCC, for shaping new diagnostic approaches and developing new therapeutic modalities to improve the prognosis of ESCC patients.

The eukaryotic translation initiation factor 3 (EIF3) is one of the largest complexes in mammalian cells. It contains 13 putative subunits named from EIF3A to EIF3M [8]. EIF3H is one of non-conserved subunits of EIF3 [9]. Expression of the *EIF3H* gene was shown to be significantly upregulated in many human cancers, including non-small cell lung cancer [10], breast cancer [11], hepatocellular carcinomas [12], colorectal cancer [13], prostate cancer [14] and osteocarcinoma [15]. A siRNA screen identifies EIF3H as a driver gene within the 8q23.3 amplicons contributing to cell growth, survival and transformation in breast cancer [11]. In lung adenocarcinoma, EIF3H functions as an oncogene by inducing EMT signaling pathway, which could be inhibited by PDCD4 [16]. Moreover, amplification of the *EIF3H* is associated with advanced stage and poor prognosis in prostate cancer [17]. Besides, the METTL3-EIF3H interface is required for enhanced translation and oncogenic transformation [18]. These observations indicate that EIF3H might have great contribution to establishing and maintaining the aggressive state of cancer. In consistence with previous studies, we also found EIF3H is overexpressed in ESCC tissues. In order to get a comprehensive understanding about the significance of EIF3H and the mechanism of its function in ESCC, we performed a liquid chromatography tandem mass spectrometry (LC-MS/MS) analysis and identified that EIF3H could interact with Snail and correlate positively with Snail expression. Furthermore, we demonstrated Snail, as the novel identified substrate of EIF3H, could be deubiquitinated and stabilized by EIF3H.

Snail is a well-known transcription factor capable of promoting epithelial-mesenchymal transition (EMT) and tumor metastasis [19], inducing cancer cell stemness and differentiation [20], contributing to cancer cell proliferation [21] and survival [22, 23], impacting on metabolism [24], suppressing immune surveillance [25] and inducing drug resistance [26]. Snail is a highly labile protein which is degraded through the ubiquitin-proteasome pathway at post-translational levels [27]. Multiple E3 ubiquitin ligases, including β-TrCP [28], FBXO11 [29], FBXL14 [30], FBXL5 [31] and SPSB3 [32], are involved in Snail ubiquitination and degradation. Protein expression is meticulously regulated by the balance between ubiquitination and deubiquitination [33], so deubiquitinating enzymes (DUBs) may play an crucial role in regulating Snail protein in the opposite direction to ubiquitination. Approximately 100 DUBs have been identified so far [34], nevertheless, a few DUBs such as DUB3 [35], OTUB1 [36], USP47 [37], PSMD14 [38], USP27X [39], USP26 [40] and USP1 [41] have been demonstrated to promote deubiquitination and stabilization of Snail. Most of these DUBs belong to cysteine proteases. The molecular mechanism underlying the post-translational regulation of Snail by other types of DUBs remains not fully understood.

Here, we identify EIF3H as a potential deubiquitinating enzyme, which is essential to deubiquitinate and stabilize Snail. Furthermore, we demonstrate that elevated expression of EIF3H in ESCC evokes enhanced malignant phenotypes of ESCC cells. Overall, our study demonstrates the importance of EIF3H in regulating ESCC tumor aggressiveness, and provides a potential therapeutic targets of ESCC.

## Methods

### Cell lines and cell culture conditions

Human esophageal squamous cell carcinoma cell lines (KYSE30, KYSE140, KYSE150, KYSE180, KYSE410, KYSE450, and KYSE510) were maintained in RPMI/1640 medium (Gibco) supplemented with 10% fetal bovine serum (Hyclone). Human esophageal epithelial cell line HET-1A and HEK293T cell line purchased from the American Type Culture Collection, were maintained in DMEM medium (Gibco) supplemented with 10% fetal bovine serum. Cells were cultured in an atmosphere of 5% CO_2_ at 37 °C.

### Antibodies and reagents

Antibodies against EIF3H (#ab251743, Abcam), Snail (#3879, CST; #14–9859-82, Thermo Scientific; #53519, Abcam), Ubiquitin (#3936, CST), Vimentin (#5741, CST), N-cadherin (#13116, CST), E-cadherin (#3195, CST), EIF3A (#3411, CST), EIF3I (#ab40745, Abcam) were used. Normal rabbit IgG (#2729, CST), anti-c-MYC agarose affinity gel (A7470, Sigma Aldrich) and anti-Flag® M2 affinity gel (A2220, Sigma Aldrich) were used for immunoprecipitation assay. MG132 (C2211, Sigma Aldrich) was dissolved in dimethyl sulfoxide. Lipofectamine 2000 (Invitrogen) was used for transfection. CCK-8 kit was purchased from Dojindo Labs.

### Plasmids and primers

Full length of human EIF3H cDNA (NM_003756.2; CCDS6319.1) was subcloned into the pLVX-IRES-puro vector (Clontech) with or without tags. Plasmids of wild-type and deletion mutants for Snail were constructed as described [38]. One short hairpin RNAs targeting Snail (shSnail: 5′-CCACTCAGATGTCAAGAAGTA-3′) and two targeting EIF3H (shEIF3H#1: 5′-CAACTCTTGGAAGAAATATA-3′; shEIF3H#2: 5′-CTGTTGCAGATAAACATGAA-3′) were constructed into the pSIH-H1 vector (System Biosciences). The following RT-qPCR primers were used in this study: EIF3H-F: 5′-CCAGCAGCAATCATTTGGGG-3′; EIF3H-R: 5′-ATATTCTCCTGCTGGCGACG-3′; Snail-F: 5′-TCGGAAGCCTAACTACAGCGA-3′; Snail-R: 5′-AGATGAGCATTGGCAGCGAG-3′. All sequences were verified by DNA sequencing.

### Cell proliferation and colony formation assays

CCK-8 assay and colony formation assay were used to evaluate cells proliferation. Briefly, cells were seeded in 96-well culture plates (Corning Life Science). At 24, 48, 72, 96 and 120 h post seeding, 10 μl CCK-8 reagent was added to the wells and incubated for 1 h. The absorbance was determined at 450 nm using a SpectraMax 190 Microplate Reader (Molecular Devices). The colony formation assay needs 1000–2000 indicated cells seeded in six-well plates and after 14 days, these cells were fixed with 1.5% crystal violet/ methanol solution and counted. For the softagar colony formation assay, 2000 established HET1A cells were mixed in 0.36% agarose culture medium as the upper layer. The bottom layer is composed of 0.75% agarose and DMEM culture medium. After an incubation for 2 weeks, the colony were stained by crystal violet and photographed.

### In vitro transwell assays

Invasion assays were performed in 24-well Milli cell chamber coated with 30 μl of Matrigel (Corning Incorporated). 3–5 × 10^4^ indicated cell lines were seeded on the coated filters in 100 μl of serum-free medium, the bottom chamber was filled with 600 μl complete culture medium. After 24 h incubation at 37 °C in 5% CO_2_, the invasive ESCC cells were stained with crystal violent. The migration assays were conducted in a similar method without coating with Matrigel.

### Immunofluorescence staining

Cells were cultivated on the μ slide VI (Ibidi) tunnels for the experiment. The cells were fixed for 15 min with 4% formaldehyde, permeabilized for 15 min with 0.5% Triton X-100 and blocked for 1 h with 5% BSA. Cells were then incubated with anti-EIF3H and anti-Snail primary antibodies overnight, and with secondary antibodies conjugated with Alexa Fluor in dark place for 1 h. The sample was then incubated with 0.5 μg/ml DAPI for 5 min. Localization of EIF3H and Snail were visualized by confocal microscopy.

### Co-immunoprecipitation (co-IP) assay

For Co-immunoprecipitation assay, 10 μM MG132 were used for incubation for 4 h before harvesting protein extraction. Whole cell lysates were mixed with anti-Flag M2 affinity gel at 4 °C for 12 h. Next, after washing the beads with RIPA buffer for five times, the immunoprecipitated protein complexes were collected for immunoblotting. Endogenous co-IP assay was performed as described above, except that total protein extraction was incubated with appropriate antibodies at 4 °C for 12 h, and then conjugated with protein A/G agarose beads (#9863, CST).

### Mass spectrometry

Vector or EIF3H-Flag plasmid was transfected and cells were cultured for 24 h. 10 μM MG132 was added into these cells for an additional 4 h. The co-IP assay and mass spectrometry procedures were performed as previously described [38]. The LC-MS/MS analysis was performed by Qinglianbio Co. Ltd. (Beijing, China).

### In vitro ubiquitination assay

Snail-FLAG together with HA-Ub and EIF3H-MYC plasmids were transfected into HEK293T, respectively. Ubiquitinated Snail and EIF3H protein were immunoprecipitated by anti-FLAG or anti-MYC affinity gel with an incubation of 10 μM MG132 for 4 h. Purified Snail and EIF3H were obtained after elution with FLAG or MYC peptides and dialysis. They were incubated in a deubiquitination reaction buffer at 30 °C for 2 h and analyzed by immunoblotting as previously described [35].

### Immunohistochemistry (IHC) analysis

To investigate the presence of EIF3H, Snail and Ki67 proteins in tumor tissues of mice, the IHC assay was performed as previously described [38]. The following antibodies were used in this assay: Ki67 with 1:200 dilution; EIF3H with 1:2000 dilution; Snail with 1:50 dilution; E-cadherin with 1: 200 dilution; N-cadherin with 1: 100 dilution. The human ESCC tissues and paired adjacent tissues used in this study were obtained from Nanjing First Hospital. These individuals did not receive any radiotherapy, chemotherapy or immunotherapy before surgery and were informed consent.

### Animal experiments

Eighteen 6 weeks old male BALB/c-nude mice were randomly divided into three groups, and subcutaneously injected with 6 × 10^5^ cancer cells into the right flank region of each mouse. After visible tumors had developed, tumor volume was measured and calculated as length × width^2^ / 2 every 2 days. Eighteen 6-week-old male SCID/Beige mice were randomly divided into three groups and injected with the KYSE150 cells (6 × 10^5^ cells each mouse) into the tail vein. One month after injection, the mice lungs were harvested, stained and weighed. These mice were purchased from Vital River (Beijing). The Institutional Animal Care and Use Committee of Chinese Academy of Medical Sciences Cancer Hospital approved these experiment procedures.

### Statistical analysis

Comparisons between two groups were performed using two-tailed Student’s *t*-test. The correlation between two groups was determined by the Pearson correlation analysis. Results from in vitro experiments shown here are representative of three independent experiments. All quantitative data are presented as mean ± s.e.m.. GraphPad Prism Version 6.01 was used for statistical analysis. A *P*-value < 0.05 was considered statistically significant.

## Results

### EIF3H is overexpressed in ESCC

**Fig. 1:**
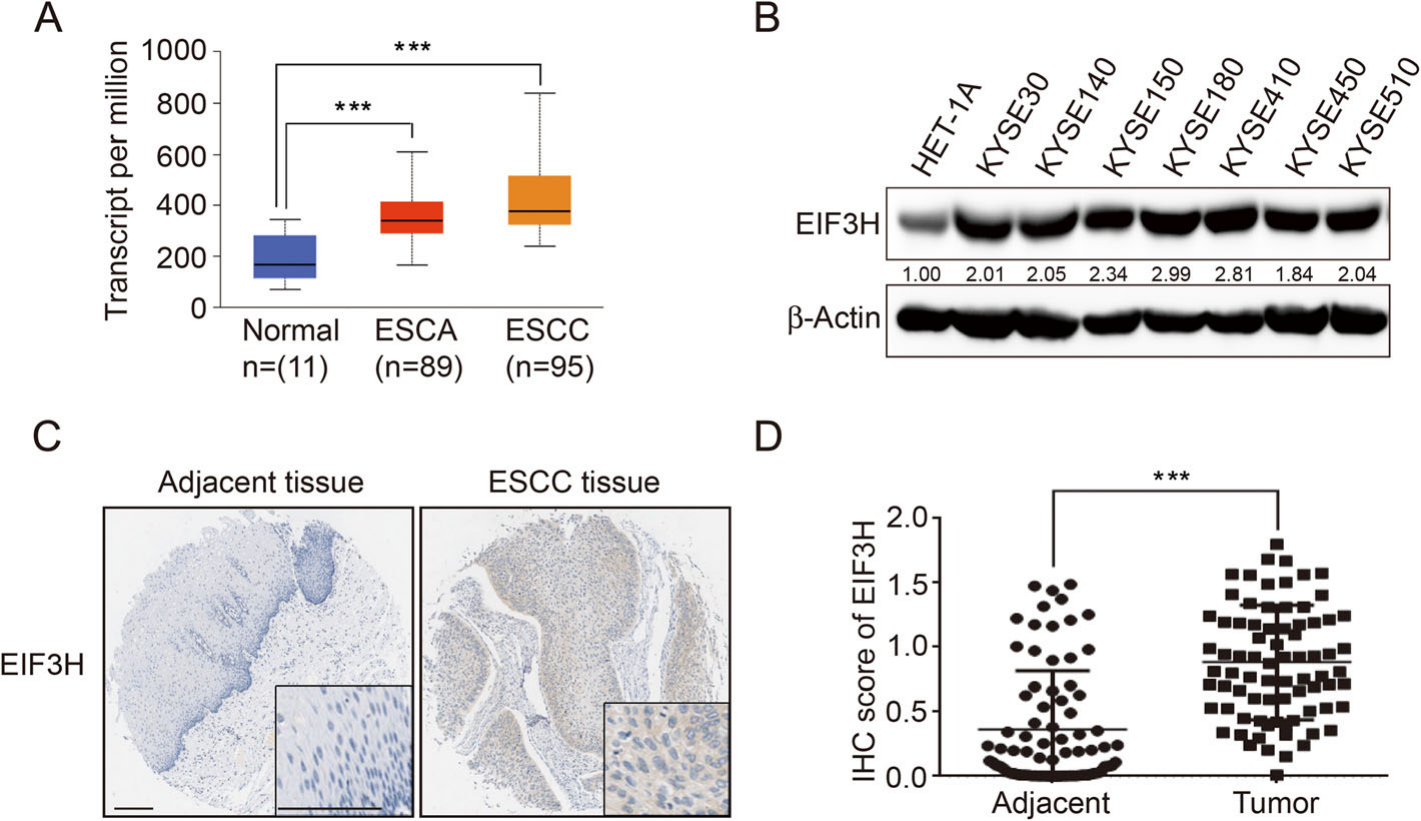
EIF3H is overexpressed in ESCC. **a** Comparison of EIF3H expression levels in esophageal cancer tissues of different subtypes with those in normal tissues. Analysis of EIF3H expression by RNA sequencing (normal *n* = 11, ESCA *n* = 89, ESCC *n* = 95, patients) from public TCGA data sets. **b** A normal human Esophageal epithelial cell line (HET-1A) and 7 ESCC cell lines (KYSE30, KYSE140, KYSE150, KYSE180, KYSE410, KYSE450, and KYSE510) were analyzed for EIF3H expression using immunoblotting. The intensity of EIF3H expression was quantified by Image J software. **c** Representative IHC images for EIF3H expression in ESCC and paired adjacent normal tissues (original magnification, × 100). Scale bars, 200 μm. **d** Comparison of the relative protein expression level of EIF3H in 79 paired ESCC and adjacent normal tissues. ***P < 0.001

We first analyzed the expression of EIF3H in esophageal cancer of TCGA data in UALCAN [42] (*n* = 195). Box plots showed that EIF3H is significantly upregulated in tumor tissues (Fig. 1a). Next, we performed the immunoblotting analysis on a panel of human esophageal epithelial cell line and ESCC cell lines. The results indicated that EIF3H is overexpressed in tumor cell lines (Fig. 1b). Using immunohistochemistry staining, we found that EIF3H expression was much higher in the ESCC tissues compared with adjacent normal esophageal epithelial tissues (Fig. 1c and d). Taken together, these results demonstrate that EIF3H is overexpressed in ESCC.

### EIF3H promotes ESCC tumorigenesis and growth in vitro and in vivo

**Fig. 2:**
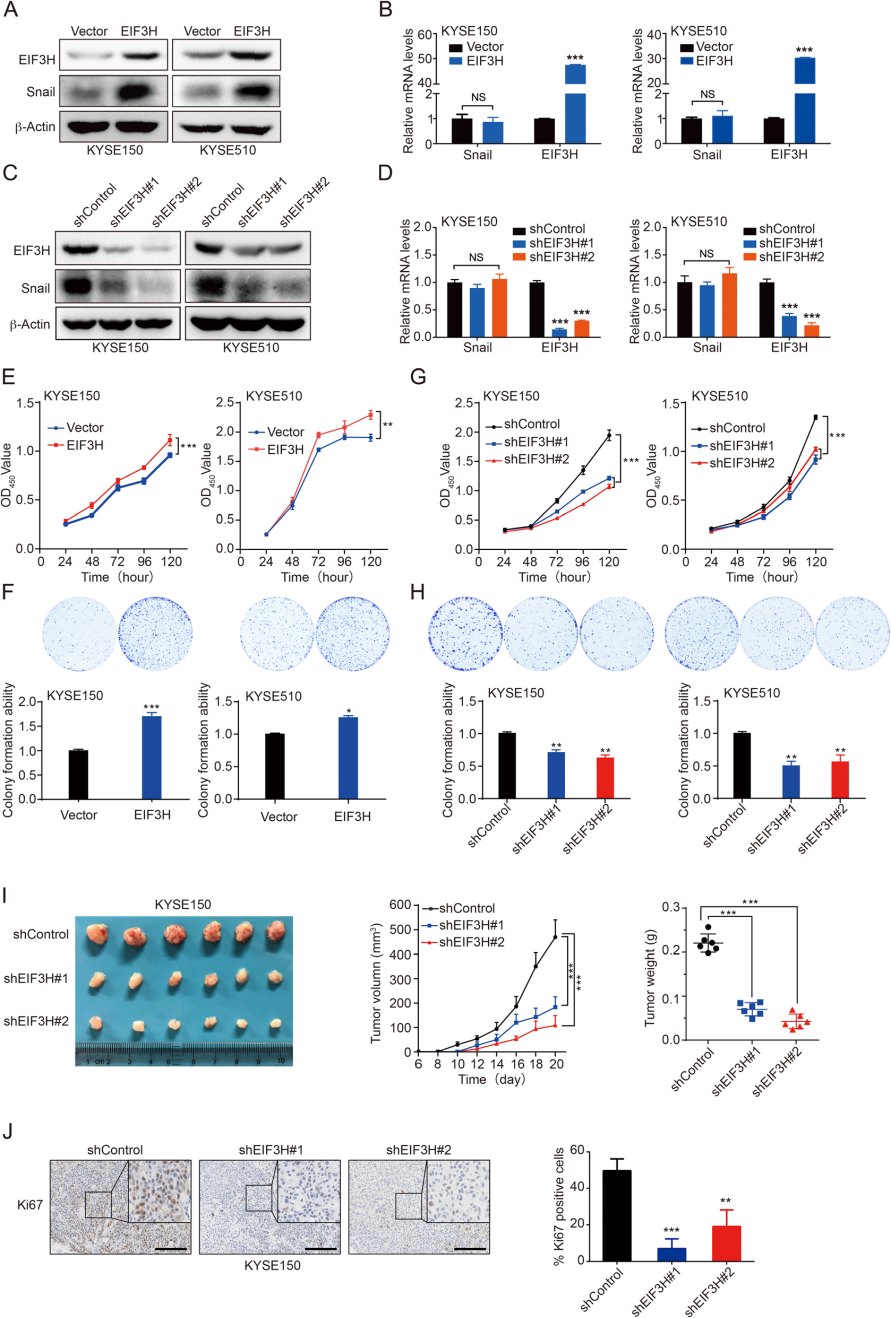
Effects of EIF3H on ESCC cell growth in vitro and in vivo. **a-d** EIF3H was stably overexpressed or knocked down in KYSE150 and KYSE510 cells. The immunoblotting showed the protein level of Snail and EIF3H (**a** and **c**), and the RT-qPCR assay was performed to detect the mRNA level of Snail and EIF3H (**b** and **d**). **e**-**h** CCK-8 or colony formation assay was performed in these established cells. Overexpression of EIF3H promoted cell proliferation and colony formation (E and F). Knockdown of EIF3H inhibited cell proliferation and colony formation. Data are shown from three independent experiments (**g** and **h**). **i** EIF3H knockdown suppressed xenograft tumor growth in vivo in mice. KYSE150 cells with or without knockdown of EIF3H were inoculated subcutaneously into the flanks of BALB/c nude mice. Tumor size was monitored for the indicated timeframe. Tumor weight was measured after the experiment. **j** IHC for Ki67 of representative areas of shControl, shEIF3H#1, and shEIF3H#2 group (left panel). Scale bars, 200 μm. Quantification of Ki67 positive cells in representative areas of shControl, shEIF3H#1, and shEIF3H#2 group (right panel). *P < 0.05; **P < 0.01; ***P < 0.001

To elucidate functional significance of EIF3H in tumor progression, we used EIF3H overexpression or knockdown KYSE150 and KYSE510 cells to detect the impact of EIF3H on cell growth. The efficacy of EIF3H overexpression or knockdown in these cells was detected by immunoblotting and RT-qPCR (Fig. 2a-d). We observed that EIF3H overexpression in both KYSE150 and KYSE510 cells increased cell proliferation in vitro (Fig. 2e and f), whereas depletion of EIF3H significantly reduced cell proliferation (Fig. 2g and h). Additionally, to detect the function of EIF3H in tumorigenesis, we established HET1A EIF3H-overexpression cell line (Fig. S1A-B). The proliferation, colony formation and malignant transformation ability were significantly improved with EIF3H overexpression (Fig. S1C-F). Moreover, in in vivo xenograft model, tumor growth and the percentage of Ki67 positivity were markedly reduced in mice injected with EIF3H knockdown KYSE150 cells compared with the controls (Fig. 2i-j). Together, these data indicate an essential role of EIF3H in cell proliferation of ESCC in the process of tumorigenesis and progression.

### EIF3H facilitates metastatic phenotypes of ESCC cells

**Fig. 3:**
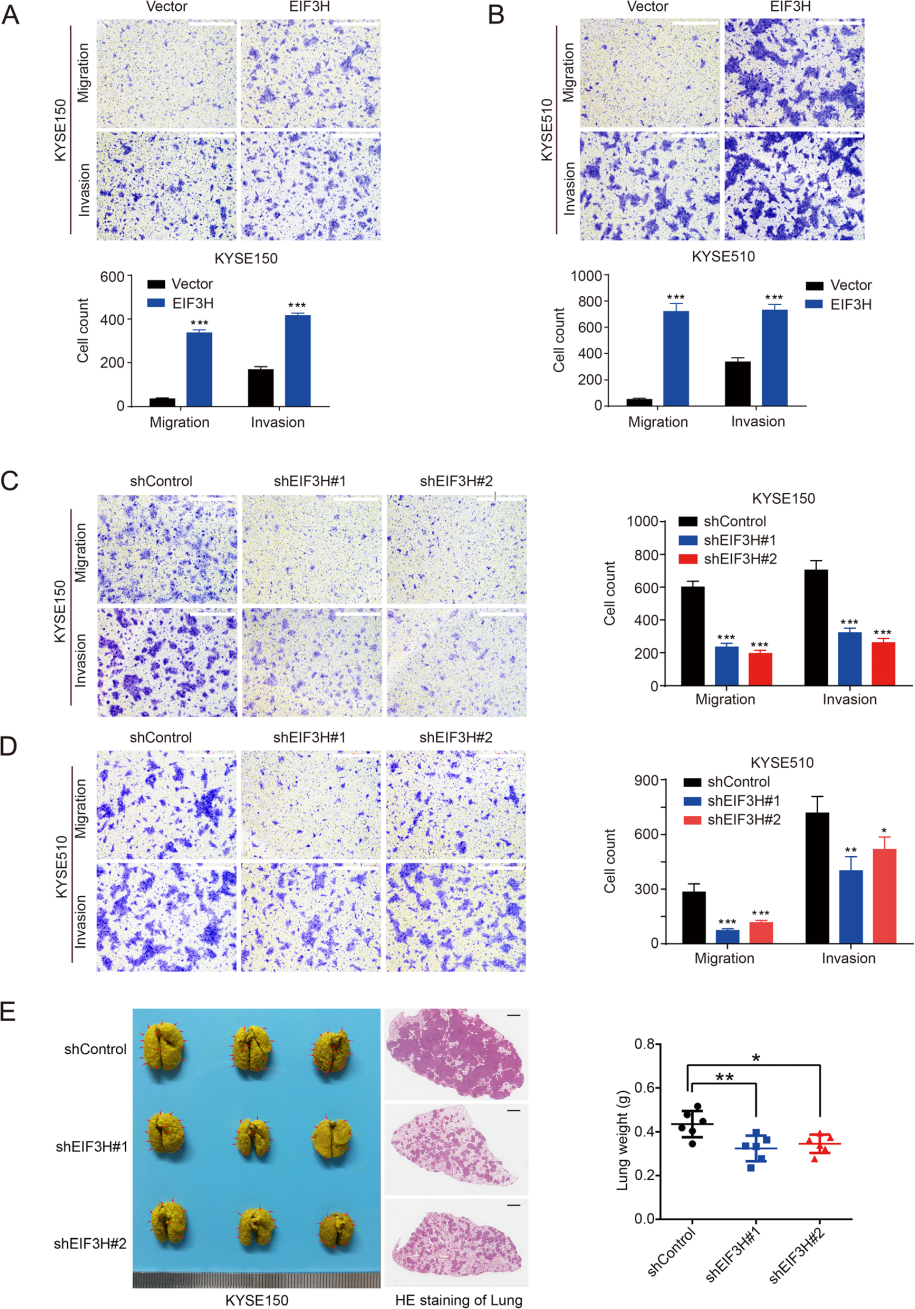
Effects of EIF3H on ESCC cell motility and tumor metastasis in vitro and in vivo**.** EIF3H was stably overexpressed (**a** and **b**) or knocked down (**c** and **d**) in KYSE150 and KYSE510 cells, in which migration and invasion assays were performed. The migratory and invasive cells were counted. Data are obtained from three independent experiments. Scale bars, 500 μm. **e** EIF3H knockdown suppressed lung metastasis in vivo in mice. KYSE150 cells with or without knockdown of EIF3H were injected into the tail vein of SCID/Beige mice (*n* = 6) and the mice lung was harvested, weighed and stained 1 month later. HE staining was performed to analyze the nodules inside the lung. The lung weight was measured after the experiment. Representative images, representative HE staining (left panel) and quantitative results of lung weight (right panel) are shown. Scale bars, 1 mm. *P < 0.05; **P < 0.01; ***P < 0.001

To further investigate the function of EIF3H on ESCC cell migration and invasion, we performed the transwell assay using ESCC cells with EIF3H overexpression or knockdown. We found that EIF3H overexpression significantly increased the abilities of migration and invasion in KYSE150 and KYSE510 cells (Fig. 3a and b). In contrast, EIF3H knockdown substantially weakened these abilities (Fig. 3c and d). To find out whether EIF3H enhances cancer metastasis ability in vivo, we then performed lung metastatic model assay. Mice injected with EIF3H knockdown cells lacked metastatic nodules in the lung tissues and the lung weight was significantly lighter than the shControl group (Fig. 3e). Taken together, these results indicate that EIF3H knockdown inhibits cell mobility and tumor metastasis in ESCC.

### EIF3H interacts with Snail

**Fig. 4:**
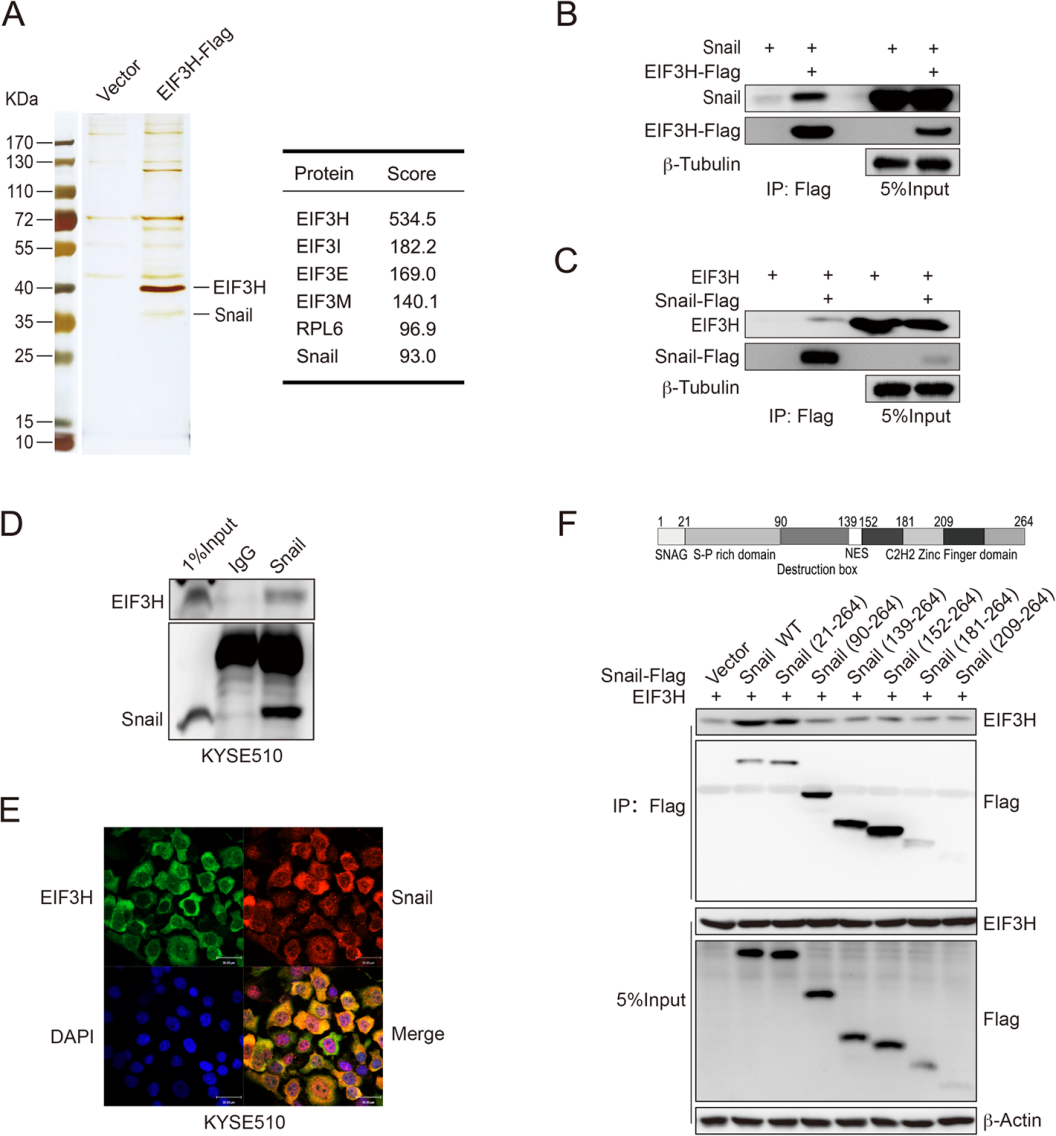
EIF3H interacts with Snail. **a** Vector or EIF3H-Flag plasmid was transfected in HEK293T cells for 24 h. After a 4 h treatment of 10 μM MG132, cell lysate was immunoprecipitated with anti-FLAG M2 beads, and the proteins interact with EIF3H were analyzed by mass spectrometry. Silver staining image was shown in the left panel. Representative proteins detected in mass spectrometry were shown in the right panel. **b** and **c** EIF3H-Flag was co-expressed with the Snail plasmid in HEK293T cells (**b**). Snail-Flag was co-transfected with the EIF3H plasmid in HEK293T cells (**c**). The co-IP assay was performed as described in (**a**). **d** Endogenous Snail was captured by anti-Snail antibody from KYSE510 cells, and the endogenous EIF3H and Snail were examined by immunoblotting. **e** Endogenous EIF3H and Snail in KYSE510 cells was detected by immunofluorescence staining. Scale bars, 30 μm. **f** The Flag-tagged empty vector, wild-type Snail or mutants plasmids were co-expressed with EIF3H plasmid. Extracts were subjected to the co-IP assay

To find out the substrate of EIF3H, we examined the proteins interacting with EIF3H using co-IP and LC-MS/MS assay (Fig. 4a). A total of 963 proteins were recognized, and among them, Snail attracted our attention because it is an EMT-associated transcription factor and ranks high on the list (Table. S1). To confirm the interaction between EIF3H and Snail, we then performed a co-IP assay with co-expressed EIF3H-Flag and Snail in HEK293T cells. After the immunoprecipitation of EIF3H, we detected an associated Snail (Fig. 4b). Then, the reciprocal co-IP assay was performed to further identify their interaction (Fig. 4c). Similar results were observed. Next, immunoprecipitation of endogenous Snail from KYSE510 cells also confirmed the interaction between endogenous Snail and EIF3H (Fig. 4d). Additional in vivo co-IP assay was performed in HEK293T and KYSE510 EIF3H-knockdown cells to test the specificity of the interaction between Snail and EIF3H (Fig. S2A-B). In the MS analysis, multiple EIF3 subunits were identified to interact with EIF3H. However, as shown in Fig. S2A-B, we only observed the immunoprecipitated EIF3H by anti-Snail antibodies but could not detect the interaction between Snail and another two EIF3 subunits EIF3A and EIF3I. In previous study, most of the EIF3H are present exclusively in the cytoplasm [43]. To shed light on how EIF3H interacts with Snail, we further determined their cellular localization by immunofluorescence analysis. The results indicated that the endogenous EIF3H mainly co-localized with the cytoplasm Snail in KYSE510 cells (Fig. 4e). We generated six truncated mutants of Snail [44] to identify which domain of Snail could interact with EIF3H. When we co-expressed EIF3H with these six truncated mutants or full-length of Snail-Flag in HEK293T cells, we found that only the full-length and the Snail truncated mutant without SNAG domain maintain their interaction with EIF3H. The truncated mutants without S-P rich domain lost their ability to interact with EIF3H (Fig. 4f). Therefore, the S-P rich domain of Snail was responsible for its interaction with EIF3H. These results revealed the interaction between EIF3H and Snail mediated by the S-P rich domain of Snail.

### EIF3H stabilizes Snail through deubiquitination

**Fig. 5:**
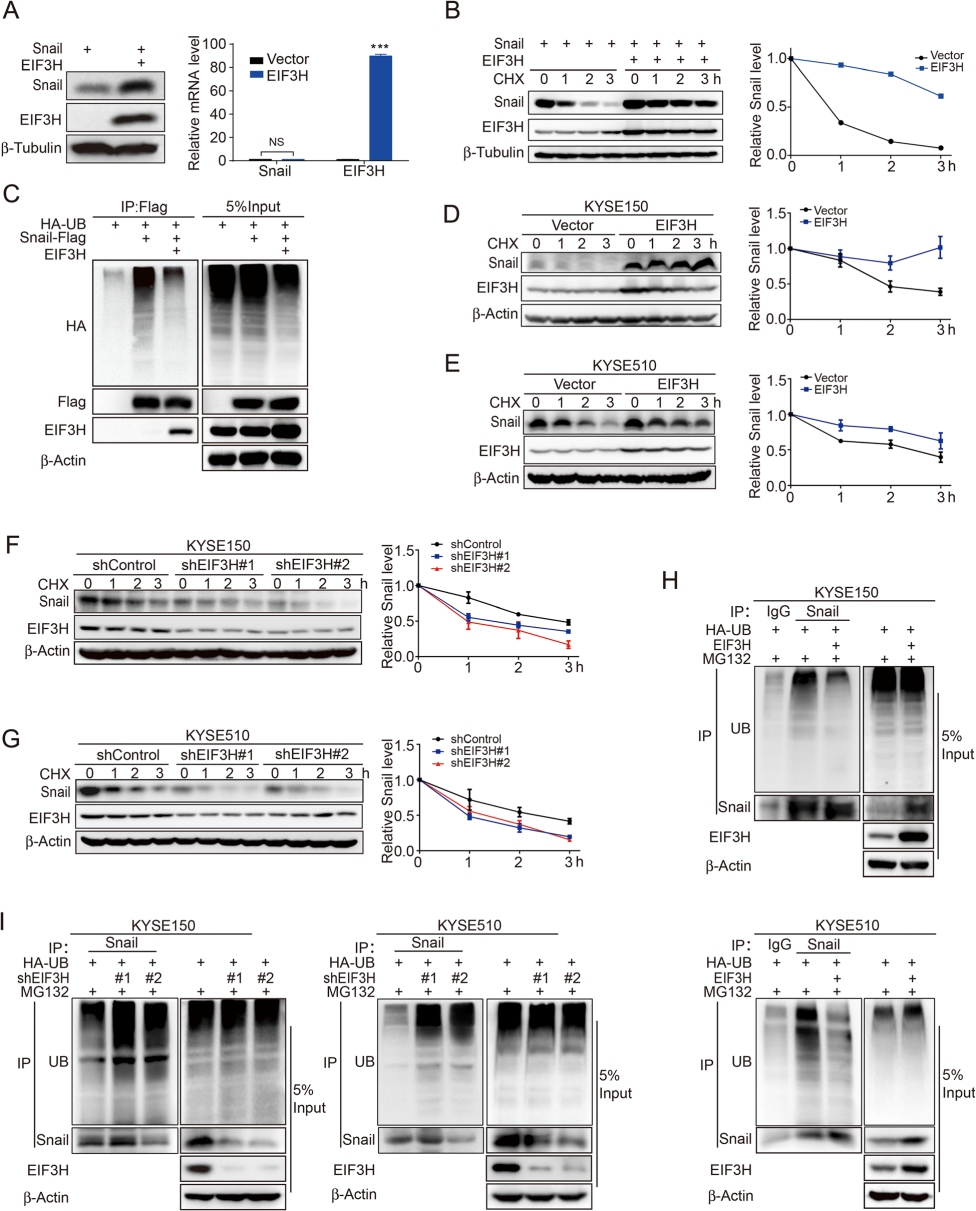
EIF3H stabilizes and deubiquitinates Snail. **a** Snail was co-expressed with vector or EIF3H in HEK293T cells. The immunoblotting showed the protein level of Snail and EIF3H (left panel) and the RT-qPCR assay was performed to detect the mRNA level of Snail and EIF3H (right panel). **b** Snail was co-expressed with vector or EIF3H in HEK293T cells. After 24 h, cells were treated with CHX (50 μg/ml) for the indicated time intervals. Expression of Snail and EIF3H was detected (left) and the intensity of Snail expression was quantified by Image J software (right). **c** Snail-Flag, HA-UB plasmids with or without EIF3H were co-transfected in HEK293T cells for 24 h. After another 4 h 10 μM MG132 incubation, the ubiquitination assay was performed to detect the poly-ubiquitination of Snail. **d** and **e** The CHX pulse-chase assay was performed on EIF3H stably overexpressed in KYSE150 and KYSE510 cells. Expression of Snail was detected (left) and quantified by the Image J software (right). **f** and **g** EIF3H was knocked down by two different shRNA in KYSE150 and KYSE510 cells. Snail half-life was analyzed by CHX pulse-chase assay, by immunoblotting (left panel) and quantified (right panel). **h** The poly-ubiquitination of Snail in KYSE150-EIF3H (upper panel) and KYSE510-EIF3H overexpression cells (bottom panel) was assessed as described in (**c**). **i** The endogenous poly-ubiquitination level of Snail in KYSE150 (left panel) and KYSE510 EIF3H knockdown cells (right panel) was detected by the deubiquitination assay as described in (**c**). Results represent mean ± s.e.m. of three independent experiments. ***P < 0.001; NS: not significant

The interaction between EIF3H and Snail suggests the ability of EIF3H to regulate Snail expression. To verify this hypothesis, we co-expressed Snail with vector or EIF3H in HEK293T cells and examined the expression of Snail and EIF3H. Results showed that EIF3H obviously upregulated the Snail protein, but did not affect the mRNA level of Snail (Fig. 5a). Subsequently, we performed a cycloheximide (CHX) pulse-chase assay to detect the function of EIF3H on Snail stability by blocking protein synthesis. EIF3H overexpression could strikingly stabilize Snail protein levels, while Snail protein degraded obviously in cells transfected with vector (Fig. 5b). We further validated whether EIF3H modulates Snail stability through deubiquitination. When co-expressed Snail-Flag with EIF3H in HEK293T cells, we found that Snail ubiquitination could be abolished by EIF3H overexpression (Fig. 5c). To further confirm the function of EIF3H on Snail stability in ESCC, we stably overexpressed or knocked down EIF3H in two ESCC cell lines, KYSE150 and KYSE510. We detected the mRNA and protein level of Snail in these cells, and revealed that the protein expression of Snail was regulated by EIF3H but the mRNA expression has no significant changes (Fig. 2a-d). Therefore, EIF3H regulates Snail expression at the posttranslational level. Then we demonstrated that EIF3H overexpression prolonged Snail half-life in the CHX pulse-chase assay (Fig. 5d and e), while endogenous Snail became unstable and degraded acceleratedly through EIF3H knockdown (Fig. 5f and g). To further extend these findings, endogenous ubiquitination assay was performed. We observed overexpression of EIF3H deubiquitinated endogenous Snail (Fig. 5h), but knockdown of EIF3H facilitated ubiquitination of endogenous Snail (Fig. 5i). Additionally, the in vitro ubiquitination assay further demonstrated that EIF3H could directly remove the ubiquitination of Snail (Fig. S2C). These results suggest that EIF3H might be a novel deubiquitinating enzyme identified to maintain Snail protein stability.

### EIF3H promotes Snail-mediated EMT in ESCC cells

**Fig. 6:**
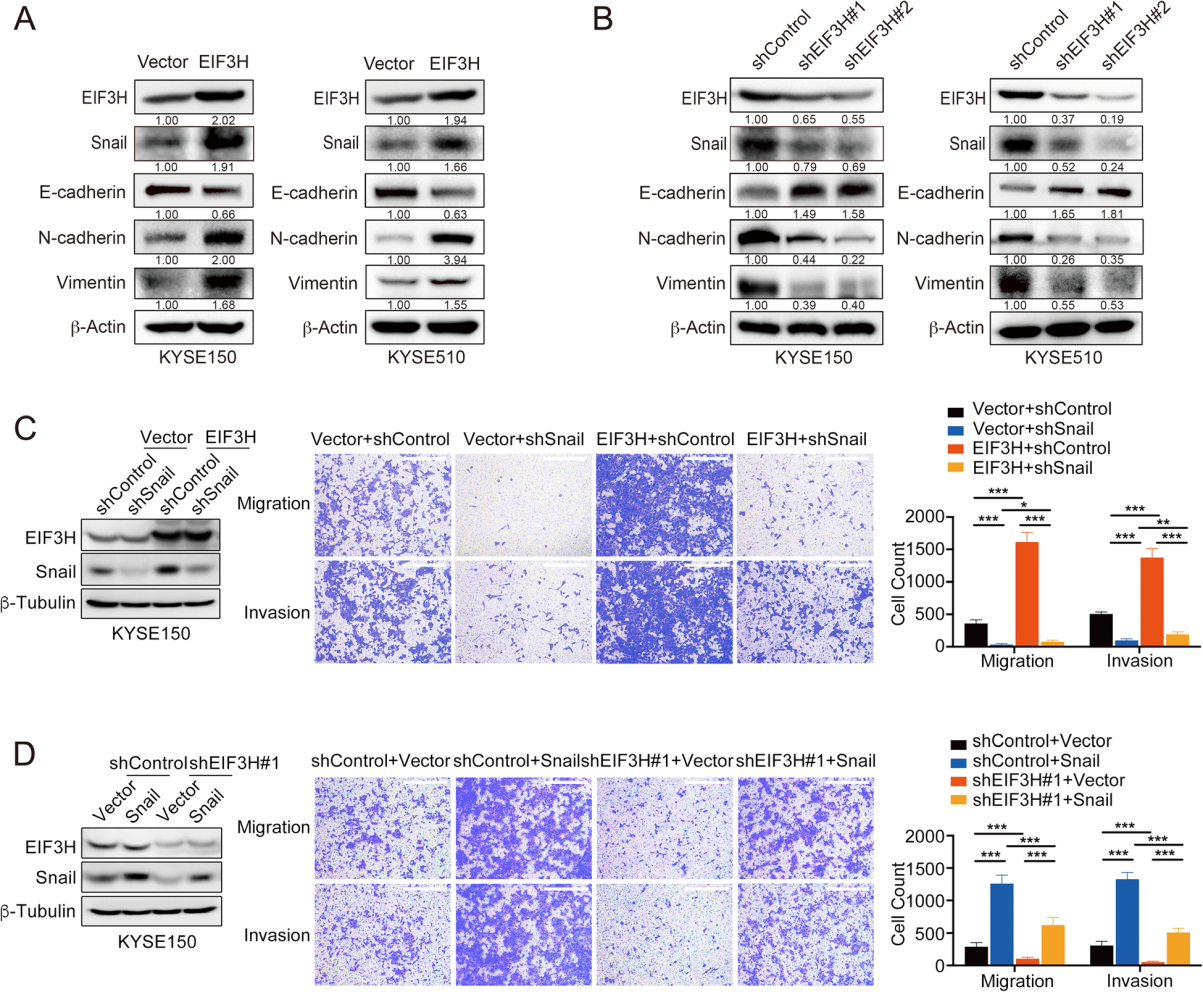
EIF3H are required for Snail-mediated EMT in ESCC cells. **a** EIF3H was stably expressed in KYSE150 and KYSE510 cells. Snail, EIF3H, E-cadherin, N-cadherin and Vimentin protein levels were analyzed by Immunoblotting in KYSE150-EIF3H (left panel) and KYSE510-EIF3H overexpression cells (right panel) and quantified by the Image J software. **b** EIF3H was knocked down by two different shRNA in KYSE150 and KYSE510 cells. The expression of Snail, EIF3H and some EMT markers, as described in (**a**), were analyzed by Immunoblotting in KYSE150-shEIF3H (left panel) and KYSE510-shEIF3H cells (right panel) and quantified by the Image J software. **c** and **d** Snail was knocked down in KYSE150-EIF3H overexpression or control cells (**c**) or overexpressed in KYSE150-shEIF3H#1 or shControl cells (**d**). The Snail and EIF3H expression were analyzed by immunoblotting (left panel). The migration and invasion ability of these cells was analyzed by the transwell assay (middle panel), and the migratory and invasive cells were counted (right panel). Scale bars, 500 μm

Considering that Snail is a pivotal EMT-related transcription factor [45], we investigated the expression of Snail and EMT-related markers in EIF3H overexpression cells. As shown in Fig. 6a, EIF3H expression increased Snail protein level. Meanwhile, these results showed that EIF3H overexpression in KYSE150 and KYSE510 cells led to the decrease of the protein level of epithelial markers (E-cadherin) as well as the increase of mesenchymal markers (N-cadherin and Vimentin). Next, we replicated these experiments in EIF3H knockdown cells. The results exhibited that knockdown of EIF3H inhibited Snail protein level, and upregulated E-cadherin protein, whereas downregulated expression of N-Cadherin and Vimentin (Fig. 6b). Snail was simultaneous knocked down in KYSE150-EIF3H overexpression or control cells. The effect of EIF3H in promoting the migration and invasion ability of KYSE150 cells was significantly reversed by the deletion of Snail (Fig. 6c). Conversely, ectopic expression of Snail in KYSE150-shEIF3H#1 cells largely increased the number of migratory and invasive cells and almost neutralized the function of the knockdown of EIF3H (Fig. 6d). Collectively, these results indicate that EIF3H could promote Snail**-**mediated EMT in ESCC cells.

### EIF3H and Snail levels correlate positively in ESCC

**Fig. 7:**
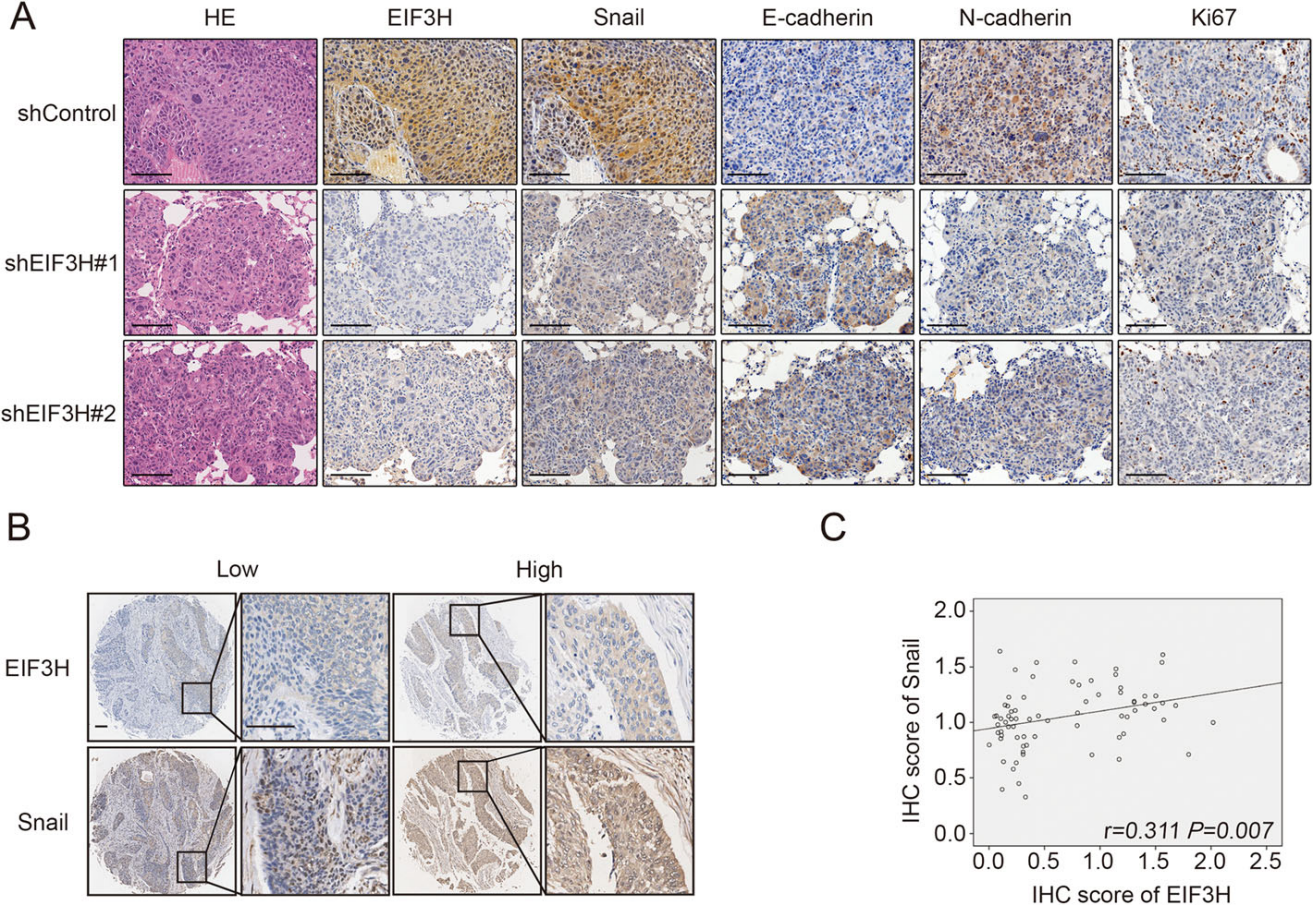
EIF3H and Snail levels positively correlate in ESCC. **a** Representative HE staining, EIF3H, Snail, E-cadherin, N-cadherin and Ki67 immunohistochemistry staining in lung tissues of shControl, shEIF3H#1 and shEIF3H#2 groups described in Fig. 3 (**e**). Scale bars, 100 μm. **b** Representative staining of EIF3H and Snail in ESCC samples. Scale bars, 100 μm. **c** The positive correlation was obtained in ESCC samples between EIF3H and Snail protein expression

Having established that EIF3H tightly regulates Snail in ESCC cells**,** we next explored our findings in the lung tissue in the tail-vein injection model in which EIF3H knockdown KYSE150 cells compared with the controls was used (Fig. 7a). The results demonstrated that knockdown of EIF3H suppressed the expression of Snail and Snail-mediated EMT switch in vivo. The percentage of Ki67 positive tumor cells in lung tissues of shControl group is also slightly higher than the EIF3H knockdown groups. To further extend the present findings in clinical, we subsequently detected the expression of EIF3H and Snail in the tissue microarray containing 73 paired ESCC tissues to investigate the clinic correlation (Fig. 7b). In concordance with our findings in xenograft model, the expression levels of EIF3H were positively correlated with Snail expression in the ESCC tissue samples (Fig. 7c). Thus, these data in ESCC tissues validated our observations in cell lines and in animal models, lending further support to our hypothesis that the EIF3H is required for the stabilization of Snail and the aggressiveness of ESCC.

## Discussion

Previous research demonstrated that EIF3H is involved in multiple cancers. Overexpression of EIF3H promotes cancer cell growth [14], results in a malignant phenotype [46], and serves as a prognostic marker of recurrence and metastasis [12, 47]. However, little is known about the role of this molecule in ESCC. Thus, this study aimed to elucidate the function of EIF3H and the molecular mechanism underlying the functional relevance in ESCC.

Validated by the expression difference in human ESCC clinical samples and their adjacent tissues, we observed that EIF3H is highly expressed in ESCC. Additionally, an oncogenic role for EIF3H in tumor progression and metastasis in this research is convincingly demonstrated in vitro and in vivo, which is in alignment with previous studies [15, 48, 49]. EIF3H depletion in ESCC cells significantly decreased the ability of cell proliferation and mobility, examined by CCK8 assay, colony formation assay and transwell assay in vitro and xenograft and tail-vein lung metastatic mouse models in vivo. In this study, we verified that EIF3H is involved in different processes of tumor tumorigenesis and progression. The increase of proliferation rate is significantly higher in EIF3H-overexpression HET1A cells than that of EIF3H-overexpression KYSE150 and KYSE510 cells but is in nearly the same proportion of EIF3H knockdown KYSE150 and KYSE510 cells. In clinical samples, EIF3H levels in primary tumors are significantly higher than their adjacent normal samples. Taken together, EIF3H might be more essential in the process of tumorigenesis. Besides, it also takes part in EMT process and the knockdown of EIF3H suppressed tumor metastasis in vivo. Whether EIF3H expression levels rise continuously during tumor progression? We still need more clinical samples and in vivo evidence to prove this hypothesis.

Besides, the mechanism of EIF3H mediated cell growth and migration also needs further study. EIF3H is one of the 13 subunits of the elongation initiation factor EIF3 and it connected with EIF3F and other subunits to form the functional octamer core in translational regulation and control [9]. EIF3H also belongs to the JAMM family of deubiquitinating enzymes for its putative nonconserved MPN domain. The uncharacterized deubiquitinating enzyme ability of EIF3H was verified by structural modelling analysis based on its noncanonical metalloprotease motif and in vitro deubiquitination analysis using tetra-ubiquitin cleavage assays biochemically [50]. The mis-regulation of EIF3H might disrupt this function leading to the cause of human diseases. To address this issue, we focused on the functional partners of EIF3H. We performed a co-IP assay combined with the mass spectrometry analysis and identified that an EMT-associated transcription factor, Snail could interact with EIF3H. In vivo co-IP assays in EIF3H knockdown cells were also carried out to confirm the specificity of the interaction between Snail and EIF3H, but there is no interaction with other EIF3 subunits (EIF3A and EIF3I). Additionally, EIF3H is demonstrated to be responsible for Snail deubiquitination and stability in ESCC cell lines. EIF3H significantly decreased the poly-ubiquitin level of ubiquitinated Snail in an in vitro ubiquitination assay. Therefore, we identified EIF3H as a potential deubiquitinating enzyme of Snail.

It has been reported that Snail plays an eminent role in the tumorigenesis, growth and metastasis of epithelial tumors [21, 51, 52], and one of its main mechanisms involved is the induction of EMT [53]. EMT is a cellular process accompanied by the loss of epithelial phenotypes and the gain of mesenchymal features. EMT is integral in cell development, stemness and contributes pathologically to cancer progression [54]. Mounting evidences also suggest that the acquisition of cancer invasiveness is correlated to EMT [55, 56] and tumor metastasis is the major cause of mortality in cancer patients [57]. EMT is featured by loss of E-cadherin expression and gain of N-cadherin and Vimentin [58]. Snail is a well-known transcriptional repressor of E-cadherin during EMT, and it is also shown to activate the expression of invasion-associated genes and the migratory phenotype [59]. A recent publication in lung adenocarcinoma demonstrated that EIF3H overexpression could also induce EMT signaling pathway [16]. Our results exhibited that ectopic expression of EIF3H promoted ESCC metastasis with the increase of Snail, N-Cadherin and Vimentin, and decrease of E-cadherin, while knockdown of EIF3H exerted an opposite effect. Knockdown of Snail strikingly reversed the promoting effects of ectopic expression of EIF3H. The IHC staining of the lung metastatic nodules in tail-vein injection mice model indicated that knockdown of EIF3H correlated with Snail downregulation and it mediated EMT switch in vivo. Clinically, the levels of EIF3H were positively correlated with Snail expression. Therefore, our study demonstrates a critical EIF3H-Snail signaling axis in EMT process and tumor metastasis in ESCC. Snail expression is regulated tightly during development, and posttranslational regulation of Snail has been a frontier issue in recent years. Protein ubiquitination is a reversible posttranslational modification that regulates a broad range of biological processes [60]. The balance between ubiquitination and deubiquitination contributes to ubiquitin homeostasis, which disruption leading to tumor initiation and progression [61]. It is quite common that several deubiquitinating enzymes, working like isozymes, regulate the same substrate under different circumstances, especially in the regulation of vital transcription factors. The stability and ubiquitination of p53 are modulated by diverse deubiquitinating enzymes under different cancer types and stimuli, such as USP42, USP49, PHD3, OTUD1, OTUD5, ATXN3 [62–67]. The protein level of Snail is also reported to be precisely regulated by ubiquitination and deubiquitination [32]. Some deubiquitinating enzymes were reported to regulate its stability, such as DUB3, PSMD14, OTUB1 and USP26 [27, 36, 38, 40]. Here, we also identified Snail as a novel substrate of EIF3H. EIF3H interacts with and deubiquitinates Snail to prolong its half-life. How these enzymes collaborate with each other to control Snail expression precisely remains still unknown and needs further investigation. Meanwhile, as a potential deubiquitinating enzyme of Snail, EIF3H might be an efficient target for ESCC therapeutic treatment. Specific small molecular inhibitors targeting EIF3H would be our next research direction.

In summary, we demonstrated that EIF3H is responsible for tumorigenesis, tumor growth and metastasis of ESCC through stabilizing Snail, thus promoting the EMT phenotype in ESCC cells. We revealed the novel modulating mechanism of the Snail stability by EIF3H in ESCC. Our study also has further implications in the development of potential new therapeutic strategy for ESCC.

## Conclusions

In this study, we investigated the mechanism underlying EIF3H as an oncogene in ESCC. We found overexpression of EIF3H promotes tumorigenesis, tumor growth and metastasis in ESCC. Moreover, we found that EIF3H might be a *bona fide* deubiquitinating enzyme to stabilize Snail protein level and Snail is the novel identified substrate of EIF3H. Knockdown of EIF3H inhibits the EMT process and cell mobility induced by Snail. In ESCC clinical samples, EIF3H protein levels correlates positively with Snail levels. Our study reveals an essential EIF3H-Snail signaling axis in tumor aggressiveness in ESCC and provides EIF3H as a promising biomarker for ESCC treatment.

## Supplementary information

**Additional file 1 Table S1**. LC-MS/MS results of co-immunoprecipitation assay.

**Additional file 2 Figure S1**. EIF3H increases the proliferation and transforming potential of HET1A cell line. (A-B) EIF3H was stably overexpressed in HET1A cells and the efficacy was detected by immunoblotting and RT-qPCR. (C) CCK8 proliferation assay was performed in these established cells. (D-E) Overexpression of EIF3H promotes colony formation ability in plate and softagar colony formation assay. (F) Representative views of softagar colony formation assay. Scale bars, 500 μm.

**Additional file 3 Figure S2**. EIF3H specifically interacts and deubiquitinates with Snail. (A-B) In vivo co-IP experiments of EIF3H-knockdown HEK293T (A) and KYSE510 (B) were performed using anti-Snail antibody. EIF3H were immunoprecipitated by Snail, but EIF3A and EIF3I were not detected. (C) An in vitro ubiquitination assay of Snail-FLAG and EIF3H-MYC purified from HEK293T cells.

## Data Availability

All data generated or analyzed during this study are included in this published article and its supplementary information files.

## Abbreviations

CHX: Cycloheximide
co-IP: Co-immunoprecipitation
EIF3H: Eukaryotic translation initiation factor 3H
EMT: Epithelial-mesenchymal transition
ESCC: Esophageal squamous cell carcinoma
IHC: Immunohistochemistry
LC-MS/MS: Liquid chromatography tandem mass spectrometry
Snail: SNAIL family transcriptional repressor 1
