# Supplementary figures and images for: EIF3H promotes aggressiveness of esophageal squamous cell carcinoma by modulating Snail stability
Source: J Exp Clin Cancer Res. 2020 Aug 31;39:175. doi: 10.1186/s13046-020-01678-9 (PMC7457539; doi:10.1186/s13046-020-01678-9)

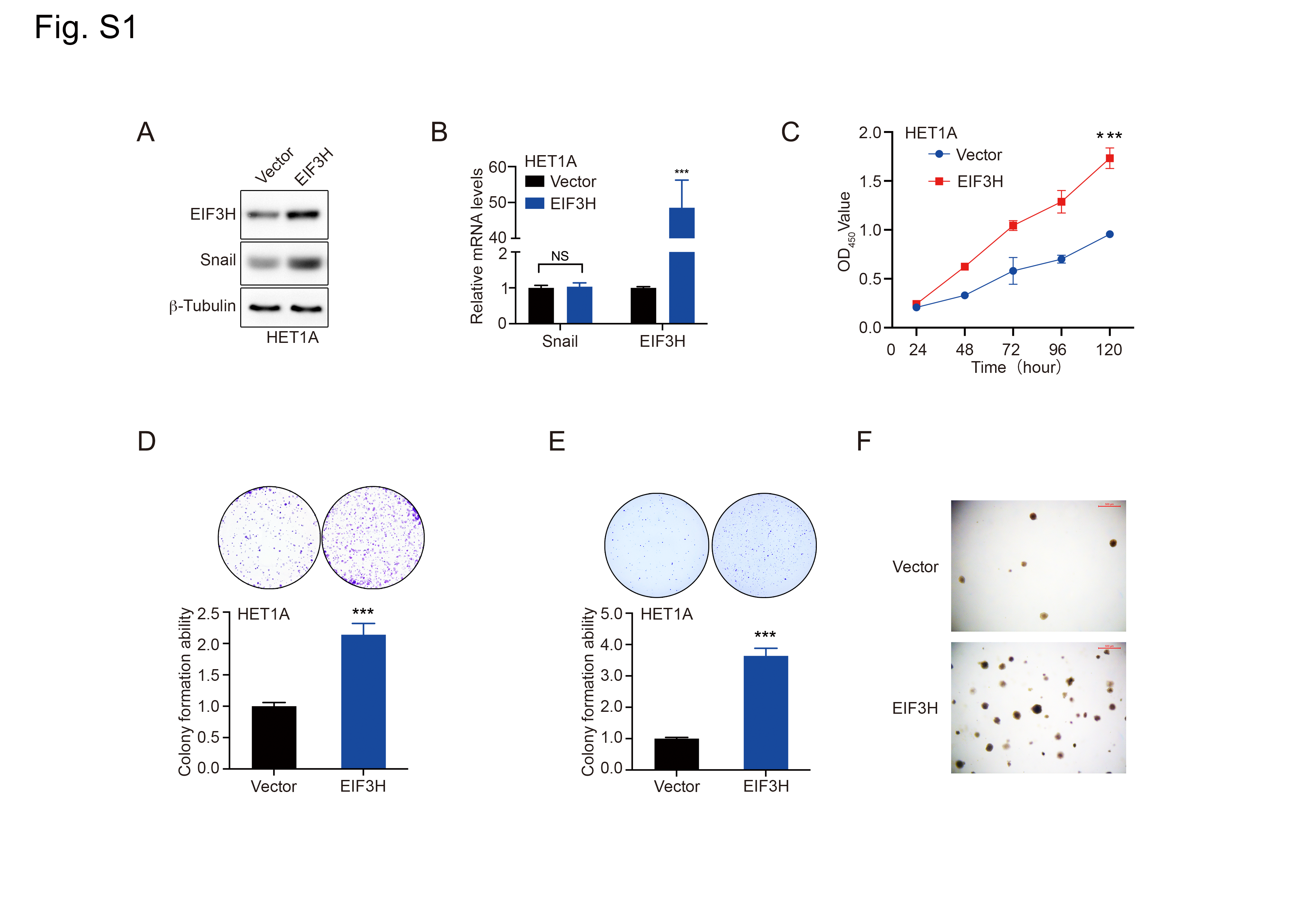

Supplement: Supplementary file 2 — Additional file 2 Figure S1. EIF3H increases the proliferation and transforming potential of HET1A cell line. (A-B) EIF3H was stably overexpressed in HET1A cells and the efficacy was detected by immunoblotting and RT-qPCR. (C) CCK8 proliferation assay was performed in these established cells. (D-E) Overexpression of EIF3H promotes colony formation ability in plate and softagar colony formation assay. (F) Representative views of softagar colony formation assay. Scale bars, 500 μm. [file 13046_2020_1678_MOESM2_ESM.tif]

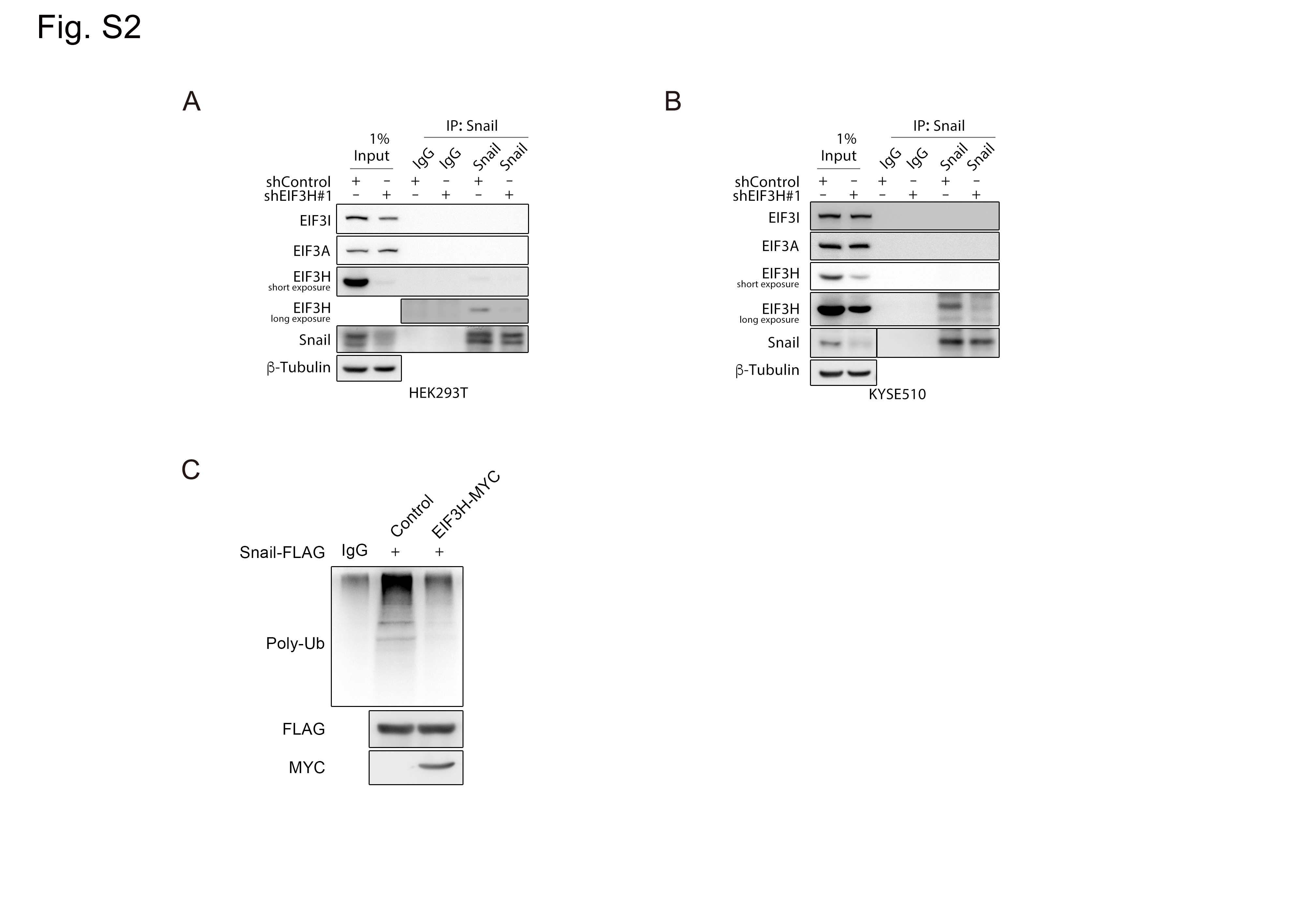

Supplement: Supplementary file 3 — Additional file 3 Figure S2. EIF3H specifically interacts and deubiquitinates with Snail. (A-B) In vivo co-IP experiments of EIF3H-knockdown HEK293T (A) and KYSE510 (B) were performed using anti-Snail antibody. EIF3H were immunoprecipitated by Snail, but EIF3A and EIF3I were not detected. (C) An in vitro ubiquitination assay of Snail-FLAG and EIF3H-MYC purified from HEK293T cells. [file 13046_2020_1678_MOESM3_ESM.tif]
